# Word Meaning Frequencies Affect Negative Compatibility Effects In Masked Priming 

**DOI:** 10.5709/acp-0186-x

**Published:** 2016-03-31

**Authors:** Andreas Brocher, Jean-Pierre Koenig

**Affiliations:** Department of Linguistics, University at Buffalo, The State University of New York, USA.

**Keywords:** masked priming, response inhibition, word recognition, negative compatibility effects, lexical ambiguity

## Abstract

Negative compatibility effects (NCEs)—that is, slower responses to targets in related than unrelated prime-target pairs, have been observed in studies using stimulus-response (S-R) priming with stimuli like arrows and plus signs. Although there is no consensus on the underlying mechanism, explanations tend to locate NCEs within the motor-response system. A characteristic property of perceptuo-motor NCEs is a biphasic pattern of activation: A brief period in which very briefly presented (typically) masked primes facilitate processing of related targets is followed by a phase of target processing impairment. In this paper, we present data that suggest that NCEs are not restricted to S-R priming with low-level visual stimuli: The brief (50 ms), backward masked (250 ms) presentation of ambiguous words (*bank*) leads to slower responses than baseline to words related to the more frequent (*rob*) but not less frequent meaning (*swim*). Importantly, we found that slowed responses are preceded by a short phase of response facilitation, replicating the biphasic pattern reported for arrows and plus signs. The biphasic pattern of priming and the fact that the NCEs were found only for target words that are related to their prime word’s more frequent meaning has strong implications for any theory of NCEs that locate these effects exclusively within the motor-response system.

## Introduction

Much of our everyday activity is grounded in automatic cognitive processes: We do not need to think about how we set one foot before another when crossing the street or how we chew when enjoying a meal. We perform these activities without conscious awareness. Not reflecting on them enables a smooth transition from cognitive processes into action. Furthermore, such automatic, procedural behavior typically goes on without much interference from simultaneously unfolding cognitive processes. One way for our cognitive system to prevent interference and avoid errors or premature actions is to *inhibit* processes that could potentially interfere with our behavior.

One well-known kind of inhibition involves negative compatibility effects (NCEs). Studies in the domain of perception and executive control have shown that the visual similarity between successively presented stimuli can sometimes slow processing down. An often-replicated example of NCEs is presented in Eimer and Schlaghecken (1998) who investigated how subliminal, masked primes affect response execution. In their first experiment, left pointing (<<), right pointing (>>), or inward or outward pointing pairs of arrows (< >; ><) were presented as primes for 16 ms and immediately backward masked for 100 ms. Masks were composed of two left and two right pointing arrows superimposed on one another. On *compatible trials*, left or right pointing primes were followed by two left (<<) or two right pointing target arrows (>>) respectively. On *incompatible trials*, primes and targets pointed in opposite directions. Inward or outward pointing primes followed by left or right pointing target arrows served as *neutral baseline trials*. Importantly, the direction of target arrows always indicated the button participants needed to press (e.g., a right pointing arrow required a right button press), thereby allowing participants to develop direct stimulus-response links (S-R links). Results showed that response times (RTs) were fastest for incompatible trials and slowest for compatible trials with neutral trials falling in between.

Numerous studies have replicated NCEs and defined boundary conditions under which NCEs can be elicited. For example, Eimer, Schlaghecken, and colleagues found that quick succession of prime and target stimuli, density of the backward mask, and (in)visibility of the prime stimuli are crucial factors for eliciting inhibition rather than positive priming (Eimer & Schlaghecken, 2002; Schlaghecken, Bowman, & Eimer, 2006; Schlaghecken & Eimer, 2000, 2002; but see Klauer & Dittrich, 2010; Lleras & Enns, 2004, 2005, 2006; Schlaghecken & Eimer, 2006; Verleger, Jaśkowski, Aydemir, Van der Lubbe, & Groen, 2004, for some counterevidence and modifications of the original claims). Another important finding is that a brief phase of positive priming precedes the inhibition, even when primes are only presented for 16 ms (Schlaghecken & Eimer, 2000). Reliable priming was elicited when the delay between prime and mask (prime-mask stimulus onset asynchrony [SOA]) was 0 ms or 32 ms and reliable inhibition when prime-mask SOAs were between 96 ms and 192 ms. This biphasic pattern was taken as a hallmark of NCEs and has been replicated in a number of studies (Jaśkowski & Przekoracka-Krawczyk, 2005; Kiesel, Berner, & Kunde, 2006; Lleras & Enns, 2005; Parkinson & Haggard, 2014; Schlaghecken & Eimer, 2002, 2004).

Interestingly, so far NCEs have almost exclusively been found when visual features were shared between primes and targets and when these features determined the response to make—that is, when S-R links were direct. This is why NCEs have been associated with S-R priming, which necessarily involves a direct link between stimulus and response. It is not yet clear whether NCEs can also be elicited with *lexical priming*, which involves the access of mental representations. In this paper, we depart from the kind of stimuli typically used in NCE tasks and examine the possibility that this kind of inhibition can occur in the absence of direct S-R links. Testing whether NCEs and the biphasic activation pattern characteristic of NCEs can depend on properties of stored representations will allow us to test two kinds of accounts that have been proposed in the literature. One kind of accounts states that NCEs are generated within the motor-response system alone and do not depend on properties of stored representations, while the other kind of accounts allows for the involvement of mental representations.

### Accounts of NCEs

Models of NCEs in S-R priming fall into two groups: Models assuming that NCEs involve the inhibition of responses and models assuming that NCEs involve the inhibition of representations. One of the first models proposing response inhibition was developed in Schlaghecken and Eimer (2002) and Eimer and Schlaghecken (2003). The authors suggest that when participants develop direct S-R links based on visual features of the presented stimuli, the NCE reflects participants’ control in perceptuo-motor links and is an example of *self-inhibition*. More specifically, they hypothesize that the NCE is generated within the motor control system and is therefore an example of response inhibition rather than stimulus inhibition. This is not to say that participants do not access any representation of a stimulus in a masked priming paradigm (Bueno & Frenck-Mestre, 2008; Draine & Greenwald, 1998; Forster & Davis, 1984; Greenwald, Abrams, Naccache, & Dehaene, 2003; Grossi, 2006; Marcel, 1983; Marslen-Wilson, Bozic, & Randall, 2008; Perea & Gotor, 1997; Perea & Rosa, 2002; Sereno & Rayner, 1992; Trueswell & Kim, 1998, for a review see Van den Bussche, Van den Noortgate, & Reynvoet, 2009) but rather that when direct perceptuo-motor links are sufficient to master a task, no higher-level, more abstract processing comes into play.

Three accounts that agree with the general claim that NCEs are generated within the motor-response system are the *object-updating account* (Lleras & Enns, 2004), the *mask-triggered inhibition account* (Jaśkowski & Przekoracka-Krawczyk, 2005), and the *ROUSE model* (Huber, 2014). However, unlike Eimer, Schlaghecken, and colleagues, these models focus on properties of the backward mask and its interaction with the prime and/or the target stimulus. More specifically, mask-related accounts assume that the mask itself causes inhibition of a previously initiated response because (1) it contains properties that are new in the visual scene and potentially important for the task (Jaśkowski, 2009; Jaśkowski & Przekoracka-Krawczyk, 2005; Jaśkowski & Verleger, 2007), (2) new features within the mask facilitate an alternative response to the prime (Lleras & Enns, 2004, 2005, 2006; see Schlaghecken & Eimer, 2006, for a response), or (3) perceptual similarity of the mask and prime leads to full saturation of the prime stimulus, impeding target processing (Huber, 2014). In all three models, response inhibition is assumed to be the result of mask-induced activation and not prime-induced inhibition of the target.

A model that assumes the inhibition of representations rather than the inhibition of responses is the *evaluation window account* (Klauer & Dittrich, 2010; Klauer, Teige-Mocigemba, & Spruyt, 2009). This account highlights participants’ ability to adapt to a task and its specific demands. According to this model, participants in a priming experiment categorize stimuli into categories that are relevant for the task, using masks as separators between stimuli. For example, in a priming experiment with arrows as stimuli, participants categorize materials into left and right pointing arrows. The model’s central claim is that participants categorize stimuli across a time window (or evaluation window), which they set to successfully manage the task. As the experiment proceeds, participants synchronize the evaluation window with the incoming stimuli to be able to quickly respond to the targets. Klauer and colleagues propose that priming occurs when primes fall within the evaluation window—that is, when primes are presented while participants prepare their response to the target. In contrast, inhibition occurs when primes are activated outside the evaluation window—that is, when primes are presented before participants prepare their response to the target.

Importantly, all models but the evaluation window account assume that direct S-R links are involved in the generation of NCEs. In other words, only the evaluation window account considers higher-level stored mental representations to also be involved. However, low-level visual stimuli like arrows and plus signs may not be ideally suited to test for the possibility that NCEs can involve higher-level, more abstract mental representations as well. This is why we tested the models we sketched above using stimuli that do not allow for the development of direct S-R links and are therefore more likely to require access of stored mental representations.

### Overview of experiments and predictions

The goal of our priming experiments was to determine (a) whether we could observe NCEs in the absence of direct S-R links and, if so, (b) whether the relative frequency of an ambiguous word’s meanings can modulate NCEs. Unlike in a typical NCE experiment, participants in our experiments did not evaluate stimuli based on their physical properties; rather, they made lexical decisions. While to date most studies in the field involved direct S-R links that were based on visual features of the stimuli, participants in our experiments needed to check a letter string onscreen against stored representations in memory to make a decision. It is important to note, however, that just like in a typical NCE experiment, primes were only briefly presented and immediately backward masked. In addition, participants were strongly encouraged to make their responses as quickly as possible. Speeded response selection was emphasized to participants, introducing some pressure on the motor-response system (see Eimer & Schlaghecken, 2002, for a discussion).

In the three experiments we report, participants judged if letter strings on the screen were existing words of English or not. They made these lexical decisions by pressing a right button on the keyboard with their right hand for “word” stimuli (e.g., *rob*) and a left button with their left hand for “non-words” (e.g., *plim*). Experiment 1 served as a control experiment and was conducted to ensure that, with clearly visible primes and targets, our materials elicited the well-established dominance effect associated with homonyms (ambiguous words with unrelated meanings): Lexical decisions to targets related to an ambiguous prime word’s more frequent, *dominant* reading are faster than neutral control whereas lexical decisions to targets related to the less frequent, *subordinate* reading of an ambiguous prime are not faster than neutral control. This effect is a typical finding in semantic priming studies and suggests that only the most frequent, dominant interpretation of an ambiguous word is initially accessed (e.g., *rob* after *bank*), while less frequent, subordinate meanings (e.g., *swim* after *bank*) do not reach their retrieval threshold before meaning selection (Duffy, Morris, & Rayner, 1988; Simpson, 1981; Simpson & Burgess, 1985; Simpson & Krueger, 1991; Tabossi, 1988). We opted for the use of homonyms with one frequent and one infrequent meaning as primes (e.g., *bank* and *cabinet*) because these words are particularly well suited to test for the involvement of representational stages in NCEs since a single prime word (e.g., *bank*) can be paired with a target word related to its more frequent meaning (*rob*) and a target word related to its less frequent meaning (*swim*).

In Experiments 2 and 3, we tested for NCEs with the materials used in Experiment 1. To that end, prime words were presented for only 50 ms and participants never responded to these primes. In both experiments, primes were immediately replaced by a subsequent pattern mask (&&&&&&&&) that remained on screen for 250 ms (Experiments 2 & 3) or 50 ms (Experiment 3). The additional inclusion of a 50 ms mask condition in Experiment 3 allowed us to test whether the biphasic pattern typical for the NCE in S-R priming (activation before inhibition) can be replicated with word stimuli.

Note that only in Experiment 1 did participants respond to prime words in addition to the target words. Although this is a procedural difference between Experiment 1 and Experiments 2 and 3, we would like to point out that dominance effects involving positive priming have also been reported for priming studies with no responses to prime stimuli (e.g., Gottlob, Goldinger, Stone, & Van Orden, 1999; Klepousniotou, Pike, Steinhauer, & Gracco, 2012; Simpson & Burgess, 1985). It is therefore unlikely that differences in (direction of) priming between Experiment 1, on the one hand, and Experiments 2 and 3, on the other, are due to the absence of prime responses in Experiments 2 and 3.

The predictions were as follows: First, if NCEs can only be elicited with S-R priming tasks, we should not observe NCEs in any of our experiments, as our materials do not encourage the development of direct S-R links. Second, if NCEs can be obtained with words and if they are generated within effector-specific motor stages alone, we should replicate the dominance effect with clearly visible prime words (Experiment 1) but not with briefly presented, masked prime words (Experiments 2 and 3). This is predicted because the dominance effect is lexical in nature and should therefore not be within the scope of NCEs under the hypothesis that NCEs are restricted to S-R-priming. Third, if NCEs can be obtained with words and be sensitive to stored properties of an ambiguous word, targets related to the dominant interpretation of a homonym might be more strongly inhibited than targets related to the subordinate meaning. This is predicted because dominant meanings are more strongly positively primed than subordinate meanings when homonyms are visible, indicating that frequency bias is part of an ambiguous word’s mental representation (Duffy et al., 1988; Simpson, 1981; Simpson & Krueger, 1991, see also Experiment 1). These results would only be compatible with the evaluation window account and would require additional assumptions for other accounts of NCEs.

## Experiment 1

### Method

#### Participants

One hundred participants of the State University of New York at Buffalo participated for course credit.^1^ All participants were monolingual native speakers of American English and reported normal or corrected-to-normal vision.

#### Materials

Because the present study investigates the processing of words with two semantically unrelated meanings one of which is significantly more frequent than the other (biased homonyms), a total of 180 words were normed for both meaning similarity and meaning dominance. Meaning similarity was defined as the degree to which speakers judge the two meanings of the ambiguous word to be semantically similar based on physical, functional, or other properties. Meaning dominance was defined as the relative frequency between the ambiguous word’s two competing interpretations. Furthermore, to ensure that participants were familiar with the subordinate meanings of ambiguous words, we also conducted a familiarity norming. No participant participated in more than one norming study.

*Similarity Norming*. In a similarity norming study, 20 monolingual native speakers of American English were presented with booklets containing 50 pairs of sentences with one content word in common. An example is provided in (1).

 1. (a) Paul wanted to deposit all his cash but the bank was already closed. 

(b) The couple went for a nice, long walk alongside the bank.

Participants were instructed to judge the meaning similarity of the two tokens of an underlined word. They were asked to base their judgments on the following questions: “Can the two meanings appear in similar contexts?” “Do they share physical or functional properties?” “Do they taste, smell, sound, or feel similarly?” “Do they behave similarly?” These questions were provided to help participants base their judgments on specific properties of the words’ meanings rather than (ad hoc) associations. Participants were instructed to provide a similarity score ranging from 1 for *not similar at all* to 7 for *the very same meaning*. In addition to including ambiguous homonyms (e.g., *bank*), the shared content words in sentence pairs also included moderately ambiguous (e.g., *cold*) and unambiguous words (e.g., *origami*) to encourage participants to use the full range of the rating scale. The 20 homonyms in sentence pairs had a mean similarity score of 1.28 (*SD* = 0.16). The fact that these similarity scores ranged from 1.1 to 1.65 confirms that the two meanings of each homonym were indeed semantically unrelated.

*Dominance Norming*. In a dominance norming study, 20 monolingual native speakers of American English were presented with booklets consisting of 48 single words, repeated on five separate lines. Each word was followed by an underscore for which participants were instructed to write down whatever came to mind. Thus, each participant provided 240 associations. Participants were encouraged to provide single words, phrases, or entire sentences. Of the 48 words in each booklet, eight were homonyms (e.g., *bank*). The remaining words were moderately ambiguous (e.g., *cold*) or unambiguous (e.g., *origami*).

For each produced association, two raters, who were trained on the task, decided whether it belonged to one of the ambiguous word’s targeted meanings (e.g.,* bank-rob* or *bank-swim*), to a different or non-comprehensible meaning (e.g., *bang*), or to either meaning (e.g., *beautiful*). Raters were instructed to only assign a particular association to one of the targeted categories (e.g., *bank-rob*) when it could not also be assigned to the competing category (e.g., *bank-swim*), even when the association was more related to one than the other. Disagreements were resolved by subsequent discussion such that a particular association was assigned to the category *different* when no agreement was reached. After resolution, overall agreement was above 90%.

For all selected items, we chose the meaning that had been produced most often as the dominant meaning. We then calculated the dominance score relative to the second, subordinate meaning. That is, we only considered the two intended readings for the calculation of the reported dominance scores so that the frequencies of the dominant and subordinate meanings always summed to 1. We computed dominance scores this way rather than compute them on the basis of all produced associations (i.e., including incomprehensible and ambiguous associations) so that they would be less susceptible to noise from unresolved raters’ disagreements or incomprehensible responses. The 20 homonyms that were selected for inclusion in our experiments had a mean dominance score of .86 for dominant and .14 for subordinate readings (both *SD*s = .1) and ranged from .67/.33 to .99/.01.

*Familiarity norming*. We finally asked 20 students from the SUNY Buffalo State College to rate the familiarity of the subordinate meanings of the ambiguous words. This was done to ensure that participants were familiar with the less frequent interpretations. Participants rated the critical word, which was underlined in a carrier sentence, on a scale from 1 for *completely unfamiliar* to 7 for *completely familiar*. Our homonyms had a mean familiarity score of 6.14 (*SD* = 0.61) and ranged from 4.65 to 6.7.

For each of the 20 homonym primes (e.g., *bank*), we selected two targets. One target word was related to the homonym’s dominant interpretation (e.g., *rob* for *bank*). The other target was related to the subordinate reading (e.g., *swim* for *bank*). We used Nelson, McEvoy, and Schreiber’s (2004) association norms to compute mean forward and backward association strength scores. Prime words and dominant-meaning related target words had a mean forward association score of .06 and prime words and subordinate-meaning related target words had a mean forward association score of .01. The difference was statistically marginal as indicated by an unpaired, two-tailed *t*-test, *t*(38) = 1.76, *p* = .086. Furthermore, the mean backward association score for homonyms and dominant-meaning related target words was .05 while the mean backward association score for ambiguous primes and subordinate-meaning related target words was .01. The difference was not significant, *t*(38) = 1.20, *p* = .245.

An unrelated baseline condition was created by pairing dominant-meaning and subordinate-meaning related target words with a semantically unrelated non-word prime. It was important to use non-words rather than unrelated legal words in the neutral baseline condition because if inhibition is generated within the motor-response system these non-word primes should lead to the preparation and subsequent inhibition of the left-hand “no” response in Experiments 2 and 3. Thus, in the baseline conditions, responses to the experimental targets, all of which required a “yes” response with the right hand, should not be suppressed. In contrast, when primes were legal words—that is, homonyms, these words should lead to the preparation and subsequent inhibition of a “yes” response with the right hand, leading to overall slower responses to subsequent word targets that also required a “yes” response with the right hand.^2^

It is important to note that our experimental design (rather typical in priming studies) led to a confound between response priming and semantic priming. That is, the condition for which semantic priming was predicted, which is for dominant-meaning related targets following an ambiguous word prime (*bank-rob*), was always response compatible, as primes and targets mapped onto the same response. However, this confound cannot explain any differences in RTs between our target stimuli because subordinate-meaning related targets and primes also always mapped onto the same response (*bank-swim*), although no semantic priming was predicted for these targets. Thus, any differences in priming or inhibition cannot be due to differences in response compatibility but will be due to differences in semantic relatedness (target related to dominant or subordinate meaning of ambiguous prime word).

Four presentation lists were constructed (see Footnote 2). Each list contained 420 stimuli (210 prime and 210 target words), leading to a total of 420 trials. Experimental trials consisted of five homonym primes paired with a dominant-meaning related target and five different homonym primes paired with a subordinate-meaning related target. This pairing was obscure to participants, however, because we employed a continuous priming format in which participants made separate responses to both prime and target words (McRae & Boisvert, 1998). Furthermore, each presentation list contained five dominant-meaning and five subordinate-meaning related targets that were preceded by a semantically neutral non-word (baseline trials). Filler prime-target pairs included words and non-words. Non-words also included pseudohomophones (e.g., *grane*) as distractors to further disguise our experimental manipulation and increase task difficulty (Azuma & Van Orden, 1997; Borowsky & Masson, 1996). The ratio between words and non-words was 1:1 (for primes and targets) and no prime or target word was repeated within a list.

### Procedure

**Figure 1.**
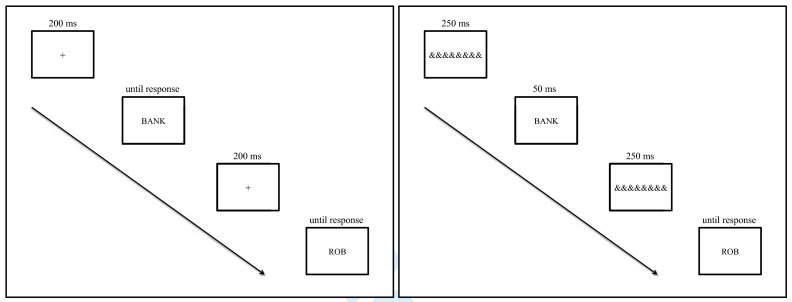
Illustration of materials and trial structure in experiment 1 (left box) and experiment 2 (right box).

We used a continuous priming procedure to obscure relationships between primes and targets (see Hutchison, 2003). Participants were tested individually and completed the experiment in one single session. Each trial began with a fixation cross in the center of the screen. This cross remained on the screen for an ISI of 200 ms and was then replaced by a letter string. All words appeared as separate trials and required a response by the participant. An illustration of a trial is provided in Figure 1.

Participants were instructed to decide as quickly and as accurately as possible, whether or not the letter string on the screen (primes and targets) was a word of English. They were instructed to press the “yes” button with their right hand if they thought the letter string was a word, and the “no” button with their left hand if they decided that it was not a word. After a lexical decision was made, the fixation cross for the next trial appeared.

Prior to the experimental trials, participants received 24 practice trials to become familiar with the task. Feedback on speed and accuracy was provided throughout the practice session but not during the experimental trials.

#### Data Analysis

For statistical analysis, we used R (R Core Team, 2013) and lme4 (Bates, Maechler, Bolker, & Walker, 2014) to conduct linear mixed effects regression models (LMERs) on response latencies. We opted for LMERs because they have been shown to be more robust against Type-I errors than more conventional Analyses of Variance (ANOVAs), do not require RTs to be normally distributed, and allow for the inclusion of random intercepts for participants and items within one statistical model (for reviews, see Baayen, 2008, and Baayen, Davidson, & Bates, 2008). However, for completeness and comparability, we also conducted all statistical analyses using ANOVAs. Both statistical methods yielded comparable results. For the analyses on error data, we fitted generalized linear mixed effects regression models, which are a better fit for categorical data. Cohen’s *d*s were calculated using the lsr package (Navarro, 2014). We entered dominance (dominant-meaning related target vs. subordinate-meaning related target) and prime type (ambiguous prime vs. semantically neutral non-word prime) as predictors into the models and sum-coded these variables prior to analysis. We included random intercepts as well as random slopes for participants and items. Following Barr, Levy, Scheepers, and Tily (2013), random slopes were kept maximal. For all analyses reported in this paper, we determined *p*-values on the assumption that, with many observations, the *t*-distribution converges to the *z*-distribution (Baayen, 2008). Planned comparisons for dominant and subordinate target RTs following an ambiguous versus baseline prime will be reported in the text.

### Results

Three participants were excluded from the analyses due to error rates higher than 20%. All incorrect responses of the remaining participants were excluded (1.9% of data points) as well as all responses faster than 200 ms and slower than 3 s (0.5% of data points). Before statistical analysis, RTs were log-transformed using Box-Cox power transformations (Fox & Weisberg, 2011) to reduce skewness of the data. For error rate analyses, all trials were included.

**Figure 2.**
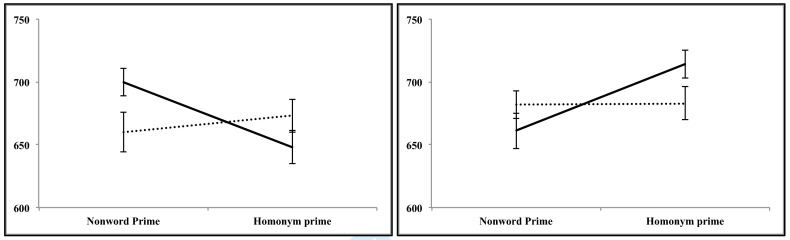
Mean lexical decision latencies and standard errors (bars around the mean) for target words in experiment 1 (left box) and experiment 2 (right box); solid lines = responses to targets related to homonyms’ dominant meaning; dotted lines = responses to targets related to homonyms’ subordinate meaning.

Results of the regression model fitted for the RT data are presented in Table 1. Mean response latencies along with the standard errors are provided in Figure 2. 

**Table 1.** Inferential Statistics for Experiments 1–3

Experiment	Main effect / Interaction	Estimate	Std. Error	*t*	*p*
1	Intercept	0,89	1,18e-05	75131,67	< .001
	Prime Type	0,56e-05	0,85e-05	0,66	.510
	Dominance × Prime Type	3,74e-05	1,70e-05	2,20	.028
2	Intercept	0,73	0,23e-05	293733,51	< .001
	Prime Type	-0,48e-05	0,11e-05	-4,18	< .001
	Dominance × Prime Type	-0,42e-05	0,20e-05	-2,08	.042
3	Intercept	0,88	0,94e-05	92650,80	< .001
	Prime Type	0,51e-05	0,50e-05	1,02	.308
	SOA × Prime Type	-2,23e-05	0,88e-05	-2,55	.011
	Dominance × Prime Type	-0,42e-05	0,94e-05	-0,44	.657
	SOA × Dominance × Prime Type	-3,32e-05	1,61e-05	-2,06	.039

*Note*. *Dominance* = dominant meaning of ambiguous word versus subordinate meaning of ambiguous word; *Prime Type* = ambiguous prime versus non-word prime; SOA (Stimulus Onset Asynchrony) = 100 ms versus 300 ms; Response times were log-transformed using Box-Cox power transformations prior to statistical analysis (see text).

Mean error rates and Cohen’s *d*s are shown in Table 2. Replicating previous research, dominant-meaning related targets elicited stronger response facilitation than subordinate-meaning related targets. This led to a significant Dominance × Prime Type interaction (see Table 1). Planned paired *t*-tests (two-tailed) revealed that RTs for dominant-meaning related targets were significantly faster after homonym primes than after semantically neutral non-word primes, *t*_1_(96) = 2.53, *p* = .046, *t*_2_(19) = 2.02, *p* = .058. In contrast, RTs for subordinate-meaning related targets did not greatly differ from that of baseline targets, *t*s < 1.5, *p*s < .1. These data replicate previous findings (Duffy et al., 1988; Simpson, 1981; Simpson & Burgess, 1985; Simpson & Krueger, 1991; Tabossi, 1988) and provide evidence that our homonyms elicit the dominance effect typical of strongly biased homonyms.

**Table 2.** Error Rates (in %) and Cohen’s *d*s for Target Words in Experiments 1–3

Experiment		Dominant	Subordinate
1	Homonym	0,6 (0,8)	2,1 (1,4)
	Non-word	2,1 (1,4)	2,9 (1,7)
	Cohen’s *d* on RTs	0,27	0,12
2	Homonym	2,8 (1,7)	4,0 (2,0)
	Non-word	3,2 (1,8)	2,0 (1,4)
	Cohen’s *d* on RTs	0,31	0,12
3 (100 ms)	Homonym	2,0 (2,0)	2,4 (2,2)
	Non-word	4,3 (2,9)	2,3 (2,2)
	Cohen’s *d* on RTs	0,26	0,12
(300 ms)	Homonym	1,7 (1,8)	2,9 (2,2)
	Non-word	3,7 (2,7)	3,0 (2,2)
	Cohen’s *d* on RTs	0,20	0,05

Note. *Dominant* = dominant meaning of ambiguous word; *Subordinate* = subordinate meaning of ambiguous word; RT = response time; for Experiment 3, the two SOA (Stimulus Onset Asynchrony) conditions (100 ms, 300 ms) are presented separately; standard errors in parenthesis.

Analyses of the error data revealed that participants responded more accurately to targets in the primed than the baseline conditions, *z* = -2.24, *p* = .025, with no reliable interaction of prime type with dominance, *z* = -1.17, *p* = .241.

Note that the slower RTs for subordinate-meaning related targets of homonyms were due to generally low baseline RTs which, in turn, were due to one particular presentation list (*M* = 674 ms, *M* = 678 ms, *M* = 626 ms, *M* = 661 ms). Even though participants in this list were overall fast readers, RTs stood out for the baseline condition of subordinate-meaning related targets. The important point here, though, is that our claims for Experiment 1 do not hinge on slower RTs than baseline for subordinate-meaning related targets.

## Experiment 2

### Method

#### Participants

One hundred and four participants of the State University of New York at Buffalo participated for course credit. All participants were monolingual native speakers of American English and reported normal or corrected-to-normal vision.

#### Materials

Materials were the same as in Experiment 1. The only difference was that, in Experiment 2, only target words required a response, leading to a total of 210 trials per presentation list.

### Procedure

Participants were tested individually and completed the experiment in one single session. The sequence of events for an individual trial closely followed the format used in masked priming involving direct S-R links. The only exception was that we used a longer stimulus onset asynchrony (SOA). We used an SOA of 300 ms, which is longer than the 116 ms SOA used by Eimer and Schlaghecken (1998). This was done on the hypothesis that the matching of a letter string against entries in the mental lexicon should take longer than the processing of an arrow or similar visual objects, and activation of masked primes need to reach a specific threshold for inhibition to occur (Schlaghecken & Eimer, 2002).

Each trial began with a forward mask consisting of eight ampersands (&&&&&&&&) that remained on the screen for an ISI of 250 ms. The forward mask was then replaced by the prime word, which was presented for 50 ms and did not require a response. The prime was immediately replaced by a backward mask, which also consisted of eight ampersands. The backward mask remained on the screen for 250 ms and was then replaced by a letter string (target), which required a lexical decision by the participant. Thus, Experiment 2 resembled the continuous priming task used in Experiment 1, with briefly presented prime words intervening between clearly visible target stimuli. An illustration of the structure of a trial is provided in Figure 1.

Participants were asked to decide as quickly and as accurately as possible, whether or not the letter string on the screen (target words) was a word of English. They pressed the “yes” button with their right hand if they thought the letter string was a word, and the “no” button with their left hand if they decided that the letter string was not a word. After a lexical decision was made, the forward mask of the subsequent trial appeared.

Prior to the experimental trials, participants received 24 practice trials to become familiar with the task. Feedback on speed and accuracy was provided throughout the practice session but not during the experimental trials. Finally, participants were told that if they noticed a flicker on the screen between the two masks (the prime), it was an artifact of the program being used and that they should disregard it.

After the experiment, participants were asked by the experimenter whether they had been aware of the primes. Although we believe that this estimation of prime visibility is sufficient to distinguish conscious and subjectively unconscious word retrieval processes, this method is a limitation of the current set of experiments (see General Discussion). After the experiment, 34 participants reported that they had noticed flickering between the forward and backward masks but that they had not paid any further attention to it. More importantly, they reported being unaware of the fact that the flickers they saw were words. Four participants reported that they had noticed words between the masks. They were even able to repeat some of the identified words back to the experimenter. Because these participants reported that they had tried to uncover the identities of the masked letter strings we excluded their data from the analysis, as their RTs were likely to reflect strategic processes.

#### Data Analysis

Data analysis was the same as for Experiment 1.

### Results

All participants performed with an accuracy of 80% or better. All incorrect responses (3.1% of data points) as well as all responses faster than 200 ms and slower than 3 s (0.3% of data points) were excluded. Before statistical analysis, RTs were log-transformed using Box-Cox power transformations. For error rate analyses, all trials were included.

Results of the regression model fitted to account for the RT data are presented in Table 1. Mean response latencies and standard errors are provided in Figure 2. Mean error rates and Cohen’s ds are reported in Table 2. As can be seen in Figure 2, dominant-meaning related targets elicited stronger inhibition than subordinate-meaning related targets. This led to a significant Dominance × Prime Type interaction (see Table 1). Planned paired *t*-tests (two-tailed) confirmed that RTs for dominant-meaning related targets were significantly slower than RTs for target words that followed semantically neutral non-word primes, *t*_1_(99) = 3.83, *p* < .001, *t*_2_(19) = 3.25, *p* = .004. In contrast, RTs for subordinate-meaning related targets did not reliably differ from RTs of baseline targets, *t*s < 1.5, *p*s > .1.

Analyses of the accuracy data showed that participants responded generally less accurately to primed targets than to targets in the baseline condition. However, no differences reached statistical significance, *z*s < 1.6, *p*s > .2.

#### Discussion

Results from Experiment 2 provide evidence that NCEs can be observed with language-specific stimuli using a similar procedure that has been used in S-R priming studies. It is widely accepted that the meanings of homonyms like *bank* and *cabinet*, which served as prime words in our Experiments 1 and 2, are stored as separate entries in long-term memory (Duffy et al., 1988; Simpson, 1981; Simpson & Burgess, 1985; Simpson & Krueger, 1991; Tabossi, 1988). One entry is associated with the word’s dominant reading (*rob*) and the other with the subordinate reading (*swim*). Importantly, this (representational) characteristic leads speakers to quickly access the homonym’s dominant meaning while the subordinate meaning becomes available more slowly or not at all, provided that the ambiguous word is clearly visible (Experiment 1). We have shown in Experiment 2 that it is also this characteristic that leads speakers to inhibit the dominant but not the subordinate meaning of a homonym when the ambiguous word is only briefly presented and immediately replaced by a mask. This suggests that the representational information that is activated with clearly visible stimuli is suppressed with immediately masked stimuli.

Note that it is unlikely that the observed priming and inhibition is due to differences in association strength because (a) both target words were only weakly associated with their ambiguous prime words and (b) the difference between dominant- and subordinate-meaning related targets was statistically marginal. Also, including association scores for each experimental prime-target pair (e.g., between *bank* and *rob*) and their interaction with prime type into the regression models did not alter the results. The crucial Dominance × Prime Type interactions remained significant, *t*s > 2, while the observed priming effects were not greatly modulated by forward or backward association strengths, *t*s < 1.3. This latter observation also renders it unlikely that our results were (greatly) influenced by strategy formation on the part of the participants. If this were the case, this should have more strongly affected RTs of target words that were more predictable (i.e., of targets with a high forward association score) and/or of targets that were more strongly backward associated to their primes (i.e., of targets with a high backward association score). Neither scenario was supported by the analyses.

We then directly compared Experiments 1 and 2 by performing a linear mixed effects model including RTs of both experiments. Experiment (Experiment 1 vs. Experiment 2), dominance (dominant-meaning related target vs. subordinate-meaning related target), and prime type (ambiguous prime vs. semantically neutral non-word prime) were included as predictors and sum-coded prior to analysis. We also again included random by-participant and by-item intercepts and random slopes into the model. As expected, the overall priming observed in Experiment 1 and the overall inhibition found in Experiment 2 led to a reliable Experiment × Prime Type interaction, β = 1.55e-05, *SE* = 0.54e-05, *t* = 2.86, *p* = .004. More importantly, we found that dominant-meaning but not subordinate-meaning related targets were primed in Experiment 1 and inhibited in Experiment 2. The Experiment × Dominance × Prime Type interaction reached significance, β = 3.09e-05, *SE* = 1.07e-05, *t* = 2.89, *p* = .004.

We also found that participants made significantly more errors in Experiment 2 than Experiment 1, *z* = 2.40, *p* = .016. Furthermore, participants made fewer errors in the primed than the unprimed conditions in Experiment 1 compared to Experiment 2, *z* = -2.26, *p* = .024. Finally, participants were marginally more accurate in the primed than in the unprimed condition when targets were dominant rather than subordinate, *z* = -1.82, *p* = .068. No other effects reached significance, *z*s < 0.1, *p*s > .0.9.

In sum, the dominant but not the subordinate interpretation of a homonym was strongly activated when the ambiguous word was clearly visible and suppressed from retrieval when the ambiguous prime word was (a) presented near the threshold of conscious awareness, (b) immediately removed from further visual processing by a subsequent mask that (c) remained onscreen for 250 ms.

Crucially, the NCE observed in Experiment 2 cannot (solely) be due to inhibition within the motor control system. If the inhibition had been generated within the motor-response system alone, responses to both the homonyms’ dominant-meaning and subordinate-meaning related targets should have been impaired because all homonymous primes were legal words of English. They all should therefore have led to the preparation and subsequent inhibition of the button press for a “word” response. Thus, subsequent targets that also required a “word” response button press (i.e., all dominant-meaning and subordinate-meaning related targets) should have elicited slower responses compared to when primes and subsequent masks inhibited a button press for non-words, which was the case for our semantically neutral baseline trials. In conclusion, data from Experiments 1 and 2 are more compatible with the view that the masked priming paradigm and stimuli we used led to the inhibition of frequent meanings of our prime words and not to the inhibition of responses.

## Experiment 3

Having shown that our homonym materials can elicit reliable inhibition with masked prime words that are presented for only 50 ms, we tried to replicate the biphasic pattern that has repeatedly been reported for low-level visual stimuli and is an important signature of NCEs (Jaśkowski & Przekoracka-Krawczyk, 2005; Kiesel, Berner, et al., 2006; Lleras & Enns, 2005; Parkinson & Haggard, 2014; Schlaghecken & Eimer, 2000, 2002, 2004). We reasoned that if the results of Experiment 2 were indeed an instance of NCEs, as they are documented in the perception and executive control literature, positive priming should be elicited at a shorter prime-target interval (50 ms) and inhibition at an intermediate prime-target interval (250 ms). Importantly, if the activation-before-inhibition processing pattern can involve conceptual domains of representation, it should be restricted to a homonym’s dominant meaning.

### Method

#### Participants

One hundred and three students of the State University of New York at Buffalo participated for course credit. All participants were monolingual native speakers of American English and reported normal or corrected-to-normal vision.

#### Materials

The same materials as in Experiments 1 and 2 were used, with the exception that four new homonyms and eight targets (four dominant and four subordinate) were added to the stimulus set to increase item power. The mean similarity score was 1.27 (*SD* = 0.16). The mean dominance score was .86 for dominant-meaning and .14 for subordinate-meaning related targets (*SD*s = .09). Finally, the mean forward association scores for items used in Experiment 3 were .05 for dominant-meaning related targets and .01 for subordinate-meaning related targets. The mean backward association scores were 0.04 and 0.01 for dominant-meaning and subordinate-meaning related targets, respectively. No difference reached significance, *t*s < 1.6; *p*s > .14.

### Procedure

The procedure was the same as in Experiment 2 with the exception that the forward and backward mask (&&&&&&&&) remained onscreen for an ISI of 50 ms in the 100 ms SOA condition and for an ISI of 250 ms in the 300 ms SOA condition. Participants either participated in the 50 ms ISI condition or the 250 ms ISI condition.

Like for Experiment 2, after the experiment participants were asked by the experimenter whether they had been aware of the primes. At debriefing, 28 participants reported that they had noticed flickering between the forward and backward masks but that they had not paid any further attention to it. Sixteen of these participants were from the 100 ms SOA condition, 12 from the 300 ms SOA condition. Three participants reported that they had noticed words between the masks and were able to repeat some of the identified words back to the experimenter. We again excluded their data from further analyses because their RTs were likely to reflect strategic processes.

#### Data Analysis

Like for Experiments 1 and 2, we fitted linear mixed effects regression models on the RT data and generalized linear mixed effects regression models on the error data. SOA (100 ms vs. 300 ms), dominance (dominant-meaning related target vs. subordinate-meaning related target), and prime type (ambiguous prime vs. semantically neutral non-word prime) were entered into the models as predictors and sum-coded prior to analysis. We also included random intercepts and random slopes for participants and items. Random slopes were again kept maximal. Planned comparisons for dominant and subordinate target RTs following an ambiguous versus a baseline prime will be reported in the text.

### Results

All participants had response accuracy higher than 80%. All incorrect responses (2.8% of data points) as well as RTs lower than 200 ms and longer than 3 s (0.6% of data points) were excluded from the analyses. Before statistical analysis, all RTs were log-transformed using Box-Cox power transformations.

**Figure 3.**
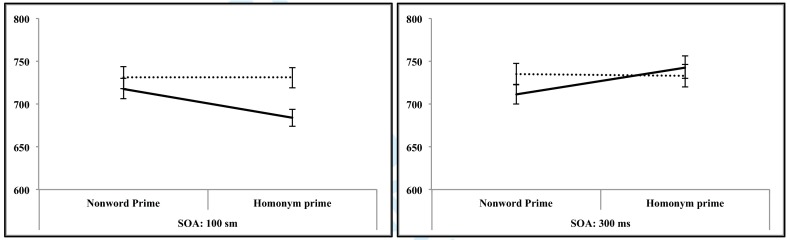
Mean lexical decision latencies and standard errors (bars around the mean) for target words in Experiment 3 for the 100 ms soA (stimulus onset Asynchrony), (left box) and the 300 ms soA condition (right box); solid lines = responses to targets related to homonyms’ dominant meaning; dotted lines = responses to targets related to homonyms’ subordinate meaning.

Results of the regression model are presented in Table 1. Follow-up statistics will be presented in the text. Mean response latencies and standard errors are provided in Figure 3. Response errors and Cohen’s *d*s are shown in Table 2. When summing across levels of SOA, we did not observe significant priming or inhibition, β = 0.51e-05, *SE* = 0.50e-05, *t* = 1.02, *p* = .308. However, replicating the biphasic pattern observed with non-verbal visual stimuli, dominant-meaning but not subordinate-meaning related targets elicited faster lexical decision times in the 100 ms SOA condition when they followed ambiguous primes than when they followed semantically neutral control primes. In contrast and replicating Experiment 2, dominant-meaning but not subordinate-meaning related targets yielded longer lexical decision times than control in the 300 ms SOA condition. This led to a significant SOA × Dominance × Prime Type interaction. Follow-up analyses confirmed that the SOA × Prime Type interaction was significant for dominant-meaning related targets, β = -3.84e-05, *SE* = 1.12e-05, *t* = -3.44, *p* < .001, but not subordinate-meaning related targets, β = -0.48e-05, *SE* = 1.34e-05, *t* = -0.36, *p* = 719. Taken together, these data indicate that the activation of a homonym’s dominant and subordinate meanings develop differently within 300 ms post homonym onset.

Importantly, like for Experiment 2, slowed responses were restricted to dominant-meaning related targets when the SOA was 300 ms. The Dominance × Prime Type interaction approached significance, β = -2.08e-05, *SE* = 1.10e-05, *t* = -1.89, *p* = .058, and the difference in RTs between the ambiguous prime and the baseline prime condition was significant for dominant-meaning, *t*_1_(49) = 3.44, *p* = .001, *t*_2_(23) = 3.02, *p* = .006, but not subordinate meaning related target words, *t*s < 1.5, *p*s > .3. Furthermore, although the Dominance × Prime Type interaction was not reliable when the SOA was 100 ms, β = 1.28e-05, *SE* = 1.44e-05, *t* = 0.89, *p* = .375, RTs (Figure 3) and Cohen’s *d* (Table 2) and show a clear and strong tendency that participants responded faster to dominant- but not subordinate-meaning related targets after ambiguous primes than after semantically neutral primes. Finally, including forward and backward association scores into the regression model did not significantly alter the results.

Analyses of the error data revealed a main effect of prime type, *z* = -2.10, *p* = .036, indicating that responses were overall more accurate in the primed than the baseline conditions. In addition, responses were overall more accurate to dominant-meaning related targets than to subordinate-meaning related targets, *z* = -2.28, *p* = .023. No other effect reached significance, *z*s < 1.1, *p*s > .2.

#### Discussion

The results of Experiment 3 suggest that the processes responsible for NCEs are very similar for tasks involving direct S-R links and tasks involving access of mental representations, as we replicated the biphasic pattern that has been reported for NCEs in S-R priming (Jaśkowski & Przekoracka-Krawczyk, 2005; Kiesel, Berner, et al., 2006; Lleras & Enns, 2005; Parkinson & Haggard, 2014; Schlaghecken & Eimer, 2000, 2002, 2004). The inhibition of a homonym’s dominant meaning observed at an intermediate SOA is absent at a short SOA. Indeed, with a short SOA and relative to the semantically neutral baseline, we observed marginal facilitation for dominant-meaning but not for subordinate-meaning related targets, although this difference was not statistically reliable in the regression model. It seems that, as with low-level visual stimuli, the NCE observed for words is preceded by a short phase of stimulus activation. This claim is supported by the significant three-way interaction between SOA, dominance, and prime type as well as the SOA × Prime Type interaction for dominant-meaning but not subordinate-meaning related targets. Replication of the biphasic activation pattern with masked words and in the absence of direct S-R links renders it unlikely that NCEs are restricted to effector-specific motor stages of processing and provides evidence that NCEs can engage more central and abstract stages of processing.

## General Discussion

In the present study we tested (a) whether (biphasic) NCEs—that is, slower responses to related (or similar) than unrelated (or dissimilar) stimuli, can arise under conditions where participants are unlikely to develop direct S-R links. To that end, we used word stimuli as well as a task that required participants to access stored representations. Our experiments capitalized on a well-known result in psycholinguistic priming studies, namely that the relative frequency of the meanings of homonyms like *bank* modulates priming. Only the most frequent meaning of a homonym prime reliably facilitates the decision whether or not a related target is a word, provided that the two meanings of the homonym have very unbalanced frequencies. If the NCE can involve the inhibition of a stored representation, we would expect a slower response than baseline only for targets related to the most frequent representation and this would show that the NCE can involve processing stages that precede the motor-response system.

Experiment 1 shows that a homonym’s more frequent but not less frequent interpretation is accessed in the absence of context. In this experiment, ambiguous words were clearly visible. In Experiment 2, homonymous prime words were presented for only 50 ms and immediately replaced by a pattern mask that lasted for 250 ms. Results were the mirror image of Experiment 1. A homonym’s more frequent but not less frequent reading was inhibited. These data were replicated for the 300 ms SOA condition of Experiment 3, although the effect was statistically less robust. In addition, data from the 100 ms SOA condition of Experiment 3 indicate that the observed inhibition is preceded by a short phase of marginal activation, replicating the biphasic pattern reported for low-level visual stimuli. Taken together, our data suggest that the NCE we elicited involved specific properties of lexical representations in long-term memory, and is therefore an example of stimulus rather than response inhibition.

### Compatibility with current models of NCEs

For NCEs in S-R priming, Eimer, Schubö, and Schlaghecken (2002)have explicitly argued against the claim that mental representations can affect NCEs. They proposed that, for stimuli that allow participants to develop S-R links exclusively based on physical properties of the stimuli—that is, direct S-R links, and for tasks that stress response speed, responses are suppressed within the motor-response system alone and reflect participants’ control in perceptuo-motor links (Eimer & Schlaghecken, 2003; Schlaghecken & Eimer, 2002).

We would first like to point out that our task (lexical decision) emphasized the need for speeded responses as well, introducing some pressure on the motor-response system. The crucial difference between our experiments and previous NCE studies lies in the kind of stimuli that were used. We pointed out above that geometrical figures such as arrows and plus signs do not constitute a strong test for the potential involvement of central processing stages in NCEs. Although participants who respond to low-level visual stimuli presumably access some representational information before that information is fed into a motor-response subsystem, visual properties within the stimuli, and not their mental representation, are most likely sufficient to develop direct S-R links. In contrast, in the experiments we report, it is very unlikely that participants could perform the task at hand by sole inspection of visual properties of the stimuli (i.e., their orthography). Instead, participants most likely checked each letter string onscreen against entries in their mental lexicon to distinguish words from non-words.

Note that we are not claiming that the self-inhibition model cannot, in principle, be extended to account for the present results. For example, one might argue that self-inhibition takes effect when direct perceptuo-motor links are sufficient to master the task at hand—that is, in tasks involving direct response specification, and that if the task requires higher-level (e.g., semantic) processing, different or additional mechanisms come into play. However, as the self-inhibition model stands, it makes no or incorrect predictions with respect to tasks and materials that do not involve direct S-R links.

Our results are also incompatible with the object-updating hypothesis put forward by Lleras and Enns (2004, 2005, 2006), the mask-triggered inhibition hypothesis proposed by Jaśkowski and colleagues (Jaśkowski, 2009; Jaśkowski & Przekoracka-Krawczyk, 2005; Jaśkowski & Verleger, 2007), and the ROUSE model entertained by Huber (Huber, 2014). All these approaches assume that visual properties within the mask or the sudden interruption of prime-related processing through the mask cause slowed responses associated with compatible targets. In Experiments 2 and 3, we used arrays of ampersands as masking objects, which do not bear close resemblance with immediately following word targets. More importantly, it is not clear how visual properties within the mask would interact with lexical processing so that only responses to dominant-meaning related targets would be inhibited.

To our knowledge, only the evaluation window account proposed by Klauer and colleagues (Klauer & Dittrich, 2010; Klauer et al., 2009) can readily explain the data reported in this study. In a typical priming experiment involving masked arrow stimuli, participants are likely to prepare their responses close to target presentation to exclude activation from potentially distracting sources. As a result, when the SOA (and therefore the mask) is long as in Experiment 2 and the 300 ms SOA condition of Experiment 3, the evaluation window does not include (activation of) the prime, leading to the observed inhibition. In contrast, when the SOA is short, like in the 100 ms SOA condition of Experiment 3, the evaluation window includes (activation of) the prime, leading to faster responses than in the neutral control condition. Thus, the evaluation window account can explain the biphasic pattern typically observed in masked priming studies (like Jaśkowski & Przekoracka-Krawczyk, 2005; Kiesel, Berner, et al., 2006; Lleras & Enns, 2005; Parkinson & Haggard, 2014; Schlaghecken & Eimer, 2000, 2002, 2004) and in Experiment 3 without alluding to the motor control system and direct S-R links. This makes this model a good candidate for explaining NCEs with both low-level perceptual stimuli, such as arrows, and higher level conceptual stimuli, such as words. The only additional assumption we need to make is that activation and inhibition of representational codes more strongly affect the dominant meaning of an ambiguous word than its subordinate meaning, a standard assumption in the ambiguity literature.

A model that shares some key features with the evaluation window model and might therefore also account for the current set of data is the *task set execution account* (Ansorge, Kunde, & Kiefer, 2014; Kiefer, Sim, & Wentura, 2015; Reuss, Kiesel, Kunde, & Hommel, 2011). This model, which has not previously been tested on NCEs, assumes that participants in an experiment adopt specific strategies or task sets to manage the task at hand. Importantly, task sets depend on the particular intentions of a participant. Evidence for this assumption comes from the finding that, in masked priming experiments with few (and/or often repeated) prime-target pairs, participants develop S-R links based on the stimuli they see in the experiment. Under these conditions, priming is restricted to prime stimuli that also appear as targets. No priming is observed for masked primes that never appear as targets. In contrast, when a stimulus set is sufficiently large, participants rely on more in-depth analyses of the stimuli onscreen. Under these conditions, priming typically transfers from clearly visible to novel masked stimuli (Kiefer et al., 2015; Kiesel, Kunde, Pohl, & Hoffmann, 2006).

What is appealing about the task set execution account is the idea that participants adopt different task sets depending on the task and instructions at hand (Ansorge & Neumann, 2005; Kiefer & Martens, 2010). In a typical masked priming experiment testing non-verbal NCEs, participants might adopt task sets based on direct S-R links developed during practice: right button press for stimuli pointing to the right; left button press for stimuli pointing to the left. No deeper analysis of the stimuli is necessary to manage the task. With larger stimulus sets, like the one used in the present study, participants might adopt task sets that require a deeper analysis of the prime stimuli: right button press for legal words; left button press for non-words. If we now assume that task sets become inhibited with a sufficiently large prime-target interval, the task set execution model could potentially account for NCEs. If we additionally assume that the discrimination of legal words and non-words leads to a semantic analysis of the presented stimuli, involving also word meaning frequencies, the task set execution account could also explain the dominance effects observed in the NCEs of the present study. Clearly, these additional assumptions need to be further tested.

### Differences between the current study and S-R priming studies

An interesting difference between the NCEs elicited in the present experiments and the NCEs elicited in former masked priming studies is that, in experiments using S-R priming, suppression of one response often led to facilitation of the alternative response. For example, a masked arrow prime that points to the left not only slows retrieval of a left pointing target arrow, it also typically facilitates retrieval of a right pointing arrow. A possible solution for the discrepancy between our data (no priming of alternative response) and the data pattern found in S-R priming studies is that inhibition-dependent facilitation might increase with increased directness of S-R links. A recent study in support of this hypothesis is Parkinson and Haggard (2014). In their study, the direction of target arrows did not indicate whether to make a left- or right-hand response, but rather whether or not to press a button. Thus, in Parkinson and Haggard’s experiments, the link between visual properties of the stimuli and the response to make was less direct. In go trials, arrows always pointed to the right. In no-go trials, arrows always pointed to the left. In free-choice trials, target arrows pointed in both directions. Crucially, inhibition of compatible responses was not always accompanied by facilitation of the alternative response, especially for free-choice trials.

Taken together, our data and results by Parkinson and Haggard (2014) suggest that the directness of the link between visual features of the stimuli and responses may determine whether or not facilitation of an incompatible response occurs when the compatible response is suppressed. If this is the case, it is not surprising that no facilitation was observed for subordinate-meaning related targets in Experiments 2 and 3 when dominant meanings were inhibited. There was no direct link between visual features of the stimuli and the responses required of participants.

A second difference between our study and previous NCE studies, which has already been pointed out above, pertains to our rather indirect estimation of unconscious processing. For practical reasons we decided on a subjective rather than an objective threshold separating conscious and unconscious processing. We assumed that participants were unable to consciously process the masked primes in Experiments 2 and 3 when they reported that they had not noticed them. However, Stenberg, Lindgren, Johansson, Olsson, and Rosen (2000) showed that even when participants report that they are unable to identify very briefly presented stimuli they can perform significantly better than chance in a subsequent forced choice task. Even though we are confident that our results reflect subjectively unconscious rather than conscious processing of masked prime words, an objective threshold of prime visibility like a forced choice task would provide a better estimate of unconscious processing than participants’ self-reports and should be employed in future studies.

### Differences between the current study and previous lexical priming studies

It is important to note that numerous studies on word recognition and sentence reading have found robust priming, including semantic priming, with briefly presented and masked primes while we elicited slowed responses (e.g., Bueno & Frenck-Mestre, 2008; Draine & Greenwald, 1998; Forster & Davis, 1984; Greenwald et al., 2003; Grossi, 2006; Kiefer & Martens, 2010; Marslen-Wilson et al., 2008; Ortells, Kiefer, Castillo, Megias, & Morillas, 2016; Perea & Gotor, 1997; Perea & Rosa, 2002; Sereno & Rayner, 1992; Trueswell & Kim, 1998; Van den Bussche, Van den Noortgate, & Reynvoet, 2009). However, most of these studies used forward masking and/or backward masking with masks of very short duration. Indeed, the short backward mask used in the short SOA conditions of Experiment 3 also yielded marginal response facilitation and thus replicated the direction of priming found in previous masked priming experiments. We surmise that responses in previous experiments that used short masks (resulting in short SOAs) were made during a phase of word activation and not word inhibition.

In conclusion, this study provides evidence that briefly presented, masked words can lead to the inhibition of the same meanings that are activated under conditions where stimuli are clearly visible and unmasked. Importantly, these results were elicited in the absence of direct S-R links. Because our data strongly suggest that the mental representation of words (in particular, the relative frequencies of their multiple meanings) can be involved in the elicitation of the NCE, we claim that a classification of the NCE as response inhibition is insufficient. Any model that locates NCEs strictly within the motor control system falls short of explaining the results we have presented, at least when moving beyond S-R priming.

## Footnotes

1 Experiments 1 and 2 each involved the manipulation of three factors, which required the creation of four lists, with 25 participants each. We only report the results of the crossing of two factors (dominance and prime type) in the present paper.

## APPENDIX A

### Materials from Experiments 1 and 2

The items are listed together with their semantic similarity scores (1 for *not similar at all* to 7 for *the very same meaning*) and relative dominance scores (frequency of dominant meaning/sense relative to the subordinate meaning/sense).

**Table:**

Prime	Similarity	Dominance	Dom target	Sub target
ball	1,35	.92	roll	dress
band	1,30	.89	drums	wrist
bank	1,45	.99	rob	swim
bar	1,25	.84	drink	wood
branch	1,30	.77	forest	store
cabinet	1,55	.88	cereal	senate
club	1,25	.91	beer	swing
coach	1,10	.91	athletic	airplane
fan	1,10	.74	sweat	praise
habit	1,40	.92	smoke	wear
horn	1,10	.72	blow	bone
jam	1,30	.95	bread	truck
navy	1,40	.83	marine	paint
pen	1,15	.98	test	farm
poker	1,25	.95	blind	metal
punch	1,10	.70	kick	bowl
racket	1,65	.94	sport	noise
right	1,30	.67	wrong	straight
seal	1,10	.81	zoo	stamp
suit	1,10	.91	coat	lawyer

Note. *Dom target* = target for dominant meaning of ambiguous prime; *sub target* = target for subordinate meaning of ambiguous prime.

### Additional Materials Used in Experiment 3

**Table:**

Prime	Similarity	Dominance	Dom target	Sub target
lap	1,32	.89	Knee	track
cast	1,10	.71	movie	nurse
chest	1,14	.80	Heart	books
port	1,48	.92	container	alcohol

Note. *Dom target* = target for dominant meaning of ambiguous prime; *sub target* = target for subordinate meaning of ambiguous prime.

## References

[R1] Ansorge U., Kunde W., Kiefer M. (2014). Unconscious vision and executive control: How unconscious
processing and conscious action control interact.. Consciousness and Cognition.

[R2] Ansorge U., Neumann O. (2005). Intentions determine the effect of invisible metacontrast-masked
primes: Evidence for top-down contingencies in a peripheral cuing
task.. Journal of Experimental Psychology: Perception &
Psychophysics.

[R3] Azuma T., Van Orden G. C. (1997). Why safe is better than fast: The relatedness of a word’s
meanings affect lexical decision times.. Journal of Memory and Language.

[R4] Baayen R. H. (2008). Analyzing linguistic data. A practical introduction to statistics using
R. Cambridge, UK: Cambridge University Press..

[R5] Baayen R. H., Davidson D. J., Bates D. (2008). Mixed-effects modeling with crossed random effects for subjects
and items.. Journal of Memory and Language.

[R6] Barr D. J., Levy R., Scheepers C., Tily H. J. (2013). Random effects structure for confirmatory hypothesis testing:
Keep it maximal.. Journal of Memory and Language.

[R7] Bates D., Maechler M., Bolker B., Walker S. (2014). lme4: Linear mixed-effects models using Eigen and S4. R package version
1.1-6..

[R8] Borowsky R., Masson M. E. J. (1996). Semantic ambiguity effects in word
identification.. Journal of Experimental Psychology: Learning, Memory, &
Cognition.

[R9] Bueno S., Frenck-Mestre C. (2008). The activation of semantic memory: Effects of prime exposure,
prime-target relationship, and task demands.. Memory & Cognition.

[R10] Draine S. C., Greenwald A. G. (1998). Replicable unconscious semantic priming.. Journal of Experimental Psychology: General.

[R11] Duffy S. A., Morris R. K., Rayner K. (1988). Lexical ambiguity and fixation times in reading.. Journal of Memory and Language.

[R12] Eimer M., Schlaghecken F. (1998). Effects of masked stimuli on motor activation: Behavioral and
electrophysiological evidence.. Journal of Experimental Psychology: Human Perception and
Performance.

[R13] Eimer M., Schlaghecken F. (2002). Links between conscious awareness and response inhibition:
Evidence from masked priming.. Psychonomic Bulletin & Review.

[R14] Eimer M., Schlaghecken F. (2003). Response facilitation and inhibition in subliminal
priming.. Biological Psychology.

[R15] Eimer M., Schubö A., Schlaghecken F. (2002). The locus of inhibition in the masked priming of response
alternatives.. Journal of Motor Behavior.

[R16] Forster K., Davis C. (1984). Repetition priming and frequency attenuation in lexical
access.. Journal of Experimental Psychology: Learning, Memory, and
Cognition.

[R17] Fox J., Weisberg S (2011). An {R} companion to applied regression (2nd ed.). Thousand Oaks, CA:
Sage..

[R18] Gottlob L. R., Goldinger S. D., Stone G. O., Van Orden G. C. (1999). Reading homographs: Orthographic, phonologic, and semantic
dynamics.. Journal of Experimental Psychology: Human Perception and
Performance.

[R19] Greenwald A. G., Abrams R. L., Naccache L., Dehaene S. (2003). Long-term semantic memory versus contextual memory in unconscious
number processing.. Journal of Experimental Psychology: Learning, Memory, and
Cognition.

[R20] Grossi G. (2006). Relatedness proportion effects on masked associative priming: An
ERP study.. Psychophysiology.

[R21] Huber D. E. (2014). The rise and fall of the recent past: A unified account of
immediate repetition paradigms.. Psychology of Learning and Motivation.

[R22] Hutchison K. A. (2003). Is semantic priming due to association strength or feature
overlap? A microanalytic review.. Psychonomic Bulletin & Review.

[R23] Jaśkowski P. (2009). Negative compatibility effect: The object-updating hypothesis
revisited.. Experimental Brain Research.

[R24] Jaśkowski P., Przekoracka-Krawczyk A. (2005). On the role of mask structure in subliminal
priming.. Acta Neurobiologiae Experimentalis.

[R25] Jaśkowski P., Verleger R. (2007). What determines the direction of subliminal
priming?. Advances in Cognitive Psychology.

[R26] Kiefer M., Martens U. (2010). Attentional sensitization of unconscious cognition: Task sets
modulate subsequent masked semantic priming.. Journal of Experimental Psychology: General.

[R27] Kiefer M., Sim E. J., Wentura D. (2015). Boundary conditions for the influence of unfamiliar non-target
primes in unconscious evaluative priming: The moderating role of attentional
task sets.. Consciousness and Cognition.

[R28] Kiesel A., Berner M. P., Kunde W. (2006). Negative congruency effects: A test of the inhibition
account.. Consciousness and Cognition.

[R29] Kiesel A., Kunde W., Pohl C., Hoffmann J. (2006). Priming from novel masked stimuli depends on target set
size.. Advances in Cognitive Psychology.

[R30] Klauer K. C., Dittrich K. (2010). From sunshine to double arrows: An evaluation window account of
negative compatibility effects.. Journal of Experimental Psychology: General.

[R31] Klauer K. C., Teige-Mocigemba S., Spruyt A. (2009). Contrast effects in spontaneous evaluations: A psychophysical
account.. Journal of Personality and Social Psychology.

[R32] Klepousniotou E., Pike G. B., Steinhauer K., Gracco V. (2012). Not all ambiguous words are created equal: An EEG investigation
of homonymy and polysemy.. Brain & Language.

[R33] Lleras A., Enns J. T. (2004). Negative compatibility or object updating? A cautionary tale of
mask-dependent priming.. Journal of Experimental Psychology: General.

[R34] Lleras A., Enns J. T. (2005). Updating a cautionary tale of masked priming:
Reply to Klapp (2005). Journal of Experimental Psychology: General, 134, 436-440..

[R35] Lleras A., Enns J. T. (2006). How much like a target can a mask be?
Geometric, spatial and temporal similarity in priming: A reply to Schlaghecken
and Eimer (2006). Journal of Experimental Psychology: General, 135, 495-500..

[R36] Marcel A. J. (1983). Conscious and unconscious perception: Experiments on visual
masking and word recognition.. Cognitive Psychology.

[R37] Marslen-Wilson W. D., Bozic M., Randall B. (2008). Early decomposition in visual word recognition: Dissociating
morphology, form, and meaning.. Language and Cognitive Processes.

[R38] McRae K., Boisvert S. (1998). Automatic semantic similarity priming.. Journal of Experimental Psychology: Learning, Memory, and
Cognition.

[R39] Navarro D. J. (2014).

[R40] Nelson D. L., McEvoy C. L., Schreiber T. (2004). University of South Florida word association, rhyme and word fragment
norms..

[R41] Ortells J. J., Kiefer M., Castillo A., Megías M., Morillas A. (2016). The semantic origin of unconscious priming: Behavioral and
event-related potential evidence during category congruency priming from
strongly and weakly related masked words.. Cognition.

[R42] Parkinson J., Haggard P. (2014). Subliminal priming of intentional inhibition.. Cognition.

[R43] Perea M., Gotor A. (1997). Associative and semantic priming effects occur at very short SOAs
in lexical decision and naming.. Cognition.

[R44] Perea M., Rosa E. (2002). The effects of associative and semantic priming in the lexical
decision task.. Psychological Research.

[R45] Ratcliff R., McKoon G. (1995). Sequential effects in lexical decision: Tests of compound-cue
retrieval theory.. Journal of Experimental Psychology: Learning, Memory, and
Cognition.

[R46] Reuss H., Kiesel A., Kunde W., Hommel B. (2011). Unconscious activation of task sets.. Consciousness and Cognition.

[R47] Schlaghecken F., Bowman H., Eimer M. (2006). Dissociating local and global levels of perceptuo-motor control
in masked priming.. Journal of Experimental Psychology: Human Perception and
Performance.

[R48] Schlaghecken F., Eimer M. (2000). A central-peripheral asymmetry in masked priming.. Perception & Psychophysics.

[R49] Schlaghecken F., Eimer M. (2002). Motor activation with and without inhibition: Evidence for a
threshold mechanism in motor control.. Perception & Psychophysics.

[R50] Schlaghecken F., Eimer M. (2004). Masked prime stimuli can bias “free” choices
between response alternatives.. Psychonomic Bulletin & Review.

[R51] Schlaghecken F., Eimer M. (2006). Active masks and active inhibition: A comment on
Lleras and Enns (2004) and on Verleger, Jaśkowski Aydemir, van der Lubbe and Groen (2004). Journal of Experimental Psychology: General, 135, 484-494..

[R52] Sereno S., Rayner K. (1992). Fast priming during eye fixations in reading.. Journal of Experimental Psychology: Human Perception and
Performance.

[R53] Simpson G. B. (1981). Meaning dominance and semantic context in the processing of
lexical ambiguity.. Journal of Verbal Learning and Verbal Behavior.

[R54] Simpson G. B., Burgess C. (1985). Activation and selection processes in the recognition of
ambiguous words.. Journal of Experimental Psychology: Human Perception and
Performance.

[R55] Simpson G. B., Krueger M. A. (1991). Selective access of homograph meanings in sentence
context.. Journal of Memory and Language.

[R56] Stenberg G., Lindgren M., Johansson M., Olsson A., Rosen I. (2000). Semantic processing without conscious identification: Evidence
from event-related potentials.. Journal of Experimental Psychology: Learning, Memory, and
Cognition.

[R57] Tabossi P. (1988). Accessing lexical ambiguity in different types of sentential
contexts.. Journal of Memory and Language.

[R58] Trueswell J. C., Kim A. E. (1998). How to prune a garden path by nipping it in the bud: Fast priming
of verb argument structure.. Journal of Memory and Language.

[R59] Van den Bussche E., Van den Noortgate W., Reynvoet B. (2009). Mechanisms of masked priming: A meta-analysis.. Psychological Bulletin.

[R60] Verleger R., Jaśkowski P., Aydemir A., van der Lubbe R. H., Groen M. (2004). Qualitative differences between conscious and nonconscious
processing? On inverse priming induced by masked arrows.. Journal of Experimental Psychology: General.

[R61] Zeelenberg R., Pecher D., de Kok D., Raaijmakers J. G. W. (1998). Inhibition from nonword primes in lexical decision reexamined:
The critical influence of instructions.. Journal of Experimental Psychology: Learning, Memory, and
Cognition.

